# Comparison of High Hydrostatic Pressure Processed Plus Sous-Vide Cooked Meat-Based, Plant-Based and Hybrid Patties According to Fat Replacement

**DOI:** 10.3390/foods11223678

**Published:** 2022-11-17

**Authors:** Rasmi Janardhanan, Mikel González-Diez, Francisco C. Ibañez, Maria Jose Beriain

**Affiliations:** Institute for Sustainability & Food Chain Innovation, Universidad Pública de Navarra, Campus Arrosadia, 31006 Pamplona, Spain

**Keywords:** fat replacing, high-pressure processing, vacuum-cooking, physicochemical trait, sensory profile

## Abstract

The impact of high-pressure processing (HPP) alone and combined with sous-vide cooking (SVCOOK) on the physicochemical and sensory traits of patties from different fat and protein matrices was evaluated. Hydro-gelled and soya emulsions were tested in meat (M), hybrid (H) and plant-based (P) patties (six formulations). M patties with pork backfat were used as reference formulation. All samples were pressurized (350 MPa, 10 min) and the HPP + SVCOOK patties were subsequently vacuum-cooked (55 °C). Significant changes (*p* < 0.05) in physicochemical parameters were detected in HPP and HPP + SVCOOK samples. Hardness reached the maximum value (11.0 N) in HPP treated P patties with soya emulsion. The HPP + SVCOOK M patties with backfat recorded the highest hardness (29.9 N). Irrespective of the fat formulations, the sensory characteristics of the HPP and HPP + SVCOOK M patties showed a well differentiated profile compared to H and P patties. The highest intensities for fatness, flavor, chewiness and the lowest for friability were recorded in HPP + SVCOOK M patties with backfat. The differences in physicochemical and sensory parameters of HPP + SVCOOK patties were minimal. Successful fat replacement using either one of the soya or hydro-gelled emulsion could be conducted in HPP + SVCOOK patties.

## 1. Introduction

In western diets, meat products serve as the major source of proteins with high biological value and micronutrients such as vitamin B_12_ and bioavailable iron [1,2]. The development of meat processing technologies and innovative products has always been a requirement in the meat industry. This industry faces concerns over environmental sustainability, climate change and animal welfare, which has led to a prominent change in dynamics relating to alternative protein products. Due to this changing diet [3,4], meat-processing companies have started exploring their alternative protein brands of products catering to the varying consumer demands. The alternative protein research and market are growing at a rather fast pace. Numerous investigations have been carried out in the area [5,6,7]. Meat protein alternatives include plant-based, cell based, fermentation based and protein extracted from insects or microalgae [8,9]. Complete replication of the muscle tissue, myofibrils and oral perception of meat using plant protein has not yet been possible. Moreover, improving the functional properties of plant proteins and their overall quality can potentially increase the market volume [5]. Recent market research suggests higher consumer acceptance for hybrid products, which are part meat and part alternative protein [10].

Meat products are an excellent source of indispensable nutrients, as mentioned above, but they also provide high amounts of saturated fatty acids and cholesterol. Research on alternative sources of fats such as plant and marine oils has been carried out [11,12,13]. The direct addition of edible oils does not promote the sensory acceptance of meat products since the normally added fats have a characteristic effect on the technological properties and the mouth feel of the products [14,15,16,17]. Oil-in-water based emulsions enriched with polyunsaturated fatty acids have exhibited promising results in fat-replaced meat products [11,18]. Polysaccharide biopolymers such as carrageenan, alginate, konjac gum, inulin and dextrin based gelled emulsions have been used as fat replacers leading to promising results [19,20,21,22]. Emulsion based fats preserve the organoleptic properties of the product. This mimics the hardness and water holding capacity of the pork backfat which is commonly used in meat products [23].

High-pressure processing (HPP) of various foodstuffs has been previously studied, and improved microbial safety and new textures preserving the technological properties of meat products are advantages of this non-thermal technology [24,25,26,27]. Research on HPP applied to different pulse proteins and starches has been reported [28,29]. HPP and thermal treatments led to similar denaturation in pulse proteins. It formed strong gels with high water holding capacity, emulsifying stability and functionalities [28]. HPP applied to starches promotes cold gelatinization [29]. Clearly, HPP in plant products has the possibility of improving the functionality of plant compounds.

Sous-vide cooking (SVCOOK) is a cooking technique used to prepare high-quality dishes. SVCOOK is a low-temperature, water bath cooking technique where the product is previously vacuum packaged. It preserves the flavor and provides a unique texture to the meat product [24]. Janardhanan et al. [30] performed a previous study on HPP and SVCOOK of meat patties and the results suggest a beneficial effect on their texture and color. Few studies have been conducted on patties manufactured with other fat and protein matrices treated with HPP plus SVCOOK. Therefore, the hypothesis that HPP combined with SVCOOK is an effective method to obtain novel patties from fat and protein matrices is proposed. The main objective of this study was to assess and compare the effect of fat formulation (pork backfat, hydrocolloid and soya emulsions) on the physicochemical and sensory characteristics of plant-based, hybrid and meat patties treated with single HPP or combined HPP + SVCOOK.

## 2. Materials and Methods

### 2.1. Emulsion and Sample Preparation

All the raw materials were locally procured. The study was conducted on patties of three different formulations (i.e., a plant-based product, a meat-based product and a hybrid product). The plant-based ingredient was Legumbreta Fina, a commercially available extruded product made from mixed flours (soy, rice and bean) and was obtained from a local manufacturer (Sanygran SL, Tudela, Spain). Plant-based samples were prepared by rehydrating the extruded product by adding 46 g of water per 100 g of raw material, followed by uniform mixing using a mixer. The hybrid product was prepared with equal parts of meat and plant-based product. The meat (*Biceps femoris*) used for the preparation of the meat-based and hybrid patties in the study was derived sustainably from Ternera de Navarra, a protected geographical indicator veal bred employing extensive cattle farming. The experiment complied with the official guidelines for the humane treatment, care and handling of animals [31].

The pork backfat used for the preparation of reference samples was ground separately (Robot Cook^®^, Robot-Coupe S.N.C., Mataró, Spain). Two formulations of emulsions were prepared, a soya based and a hydro-gelled based emulsion. Soya emulsion (*so*) was prepared using an oil mixture (olive 6.28 g; linseed 18.85 g oil/100 g), soy protein isolate (SPI) (12.02 g/100 g) and water (62.84 g/100 g) according to Gómez et al. [11] with slight changes. The SPI-water mixture was homogenized separately, followed by slow addition of the oil mixture and homogenization at 16,000 rpm (Ultra-Turrax^®^ T25basic, IKA^®^ Labortechnik GmbH & Co, Staufen, Germany). The *so* emulsion was refrigerated until use (overnight at 4 °C). Hydro-gelled emulsion (*hy*) was prepared with oil phase (olive 24 g; linseed 16 g oil/100 g), κ-carrageenan (1.5 g/100 g), water (58.45 g/100 g) and polysorbate 80 (0.05 g/100 g) based on Poyato et al. [32]. The carrageenan-water mixture and the oil phase with surfactant (polysorbate 80) were heated to 70 °C separately. Subsequently, the oil phase was slowly added to the carrageenan water mixture and homogenized. The hydro-gelled emulsion was sealed, allowed to cool to room temperature and refrigerated (4 °C) overnight to polymerize.

The patties were prepared according to Janardhanan et al. [30] by adding pork backfat (*ba*), *so* emulsion, or *hy* emulsion. The protein matrix (78.5 g/100 g), fat (20 g/100 g), salt (1.5 g/100 g), were added and blended to uniformity by a mixer (Professional Mixer Series 6, KitchenAid™, St. Joseph, MI, USA). Further, they were pressed into patties. The samples were vacuum-packaged in bags using the chamber vacuum machine (C412 Lerica, Venice, Italy). The samples were stored at 4 °C overnight until they were pressurized.

### 2.2. Experimental Design

**Figure 1:**
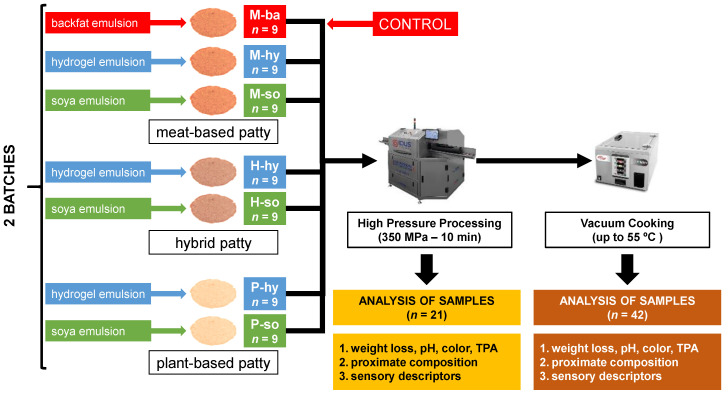
Experimental design for the sample elaboration and technological treatment according to the protein matrix (M; meat-based, H; hybrid and P: plant-based patties) and fat replacement (*ba*: backfat, *hy*: hydrogel emulsion and *so*: soya emulsion).

The patty samples were prepared for HPP and HPP + SVCOOK. The whole experiment was replicated in two batches on the same day to identify the error. A total of 126 patty patties were prepared to consist of seven formulations from meat (M), plant-based (P) and hybrid (H) protein matrices formulated with *ba* (reference), *hy* emulsion and *so* emulsion. Eighteen patties of two batches (nine each) were prepared. Forty-two patties were subjected to HPP only (HPP samples). Eighty-four patties were exposed to HPP and subsequent SVCOOK (HPP + SVCOOK samples), where one patty in each treatment was used for temperature monitoring. Figure 1 shows the flowchart for the experimental and analytical procedures.

### 2.3. Treatments

The samples were treated at 350 MPa for 10 min using an Idus machine of 10 L vessel capacity and 600 MPa of maximum pressure (Idus HPP Systems S.L.U., Noain, Spain) as described by Janardhanan et al. [30]. The pressurized samples were SVCOOK on the subsequent day. A cooking bath (Orved SV Thermo-Top, Orved S.P.A, Venice, Italy) was used for the low-temperature SVCOOK. The samples were loaded in the cooking bath when the water temperature reached 57 °C. HPP samples were cooked at a temperature of 55 °C for 15 min. Resistance temperature detector probes were used to monitor the ’product’s core temperature. Once the core reached the set temperature, the samples were taken out and immersed in cool water to stop cooking. The cooked samples were stored at 4 °C until further analysis.

### 2.4. Physical Characterization of the Emulsions

The color values and major color components of the prepared emulsions were measured using the DigiEye system (VeriVide Ltd., Leicester, UK). A rotational viscometer (Viscotester 7R, Haake, Karlsruhe, Germany) equipped with a spindle type 7 was used to calculate the apparent viscosity of the emulsions. Measures were performed at room temperature and a constant shear rate of 50 s^−1^ over 2 min. The pH of the emulsions was measured using a pH-meter fitted with a combined probe electrode (Crison Instruments S.A., Barcelona, Spain). Three replicates were performed in the parameters.

### 2.5. Proximate Analysis of Samples

Moisture [33], total protein [34], total fat [35] and total ash contents [36] of the HPP and HPP + SVCOOK processed meat, plant-based and hybrid samples were quantified.

### 2.6. Weight Loss of Samples

The weights of the individual raw samples after HPP and HPP + SVCOOK were taken and the cooking loss was calculated using the formula reported by Murphy et al. [37]:

Weight loss (g/100 g) = ((m_b_ − m_a_) × 100)/m_b_ (1)

where m_b_ and m_a_ represent the sample weights before and after HPP and HPP + SVCOOK treatments, respectively.

### 2.7. pH of Samples

The pH of the HPP samples and the combined HPP + SVCOOK samples were measured in triplicate at 25 °C [38] by means of a pH-meter (Crison Instruments S.A., Barcelona, Spain) with a combined probe electrode. The device was calibrated previously using pH buffer solutions of pH 4.01 and 7.00 at 25 °C.

### 2.8. Instrumental Color of Samples

Color coordinate (L*, a*, b*) values of the processed samples after the HPP and HPP + SVCOOK treatments were collected. A handheld spectrophotometer (Minolta 2300d, Konica Minolta Business Technol. Inc., Tokyo, Japan) was used for measuring the external color parameters, with a 52 mm Ø sphere size for D65 illuminant, 8 mm Ø measurement area, 11 mm Ø illumination area and 10° observer angle. The instrument was zero and white calibrated before use. DigiEye System (VeriVide Ltd., Leicester, UK) was also used to measure the color parameters. Six consecutive readings were recorded.

### 2.9. Instrumental Texture of Samples

Texture parameters were determined according to the method described by Mittal et al. [39]. A Texture Profile Analysis (TPA) of HPP and HPP + SVCOOK samples was conducted using a texture analyzer (TA-XT2i, Stable Micro Systems Ltd., Surrey, UK) fitted with a loadcell of 30 kg. Prior to the tests, the apparatus was calibrated with a weight of 2 kg. A cylindrical aluminum probe (25 mm Ø, 35 mm height) with a pre-test, test and post-test speed fixed as 2 mm/s was used. A trigger force of 0.06 N and a data acquisition rate of 200 pps were established. Samples (1.5 × 1.5 cm) were subjected to a two-cycle 50% compression (room temperature). The interval time between compression cycles and the compression time were set as 5 s and 3 s, respectively. Data from six consecutive measures were collected with Exponent Lite version 6.1 software (Stable Micro Systems Ltd., Surrey, UK). The parameters of hardness (N), springiness (dimensionless), cohesiveness (dimensionless) and chewiness (N) were recorded from the force-time profile.

### 2.10. Sensory Analysis of Samples

Potential assessors were screened to determine availability and interest from the staff of Universidad Pública de Navarra. Full disclosure of the study requirements, risks and ability to withdraw from the study at any time were ensured and verbal consent was attained. The panelists were verbally informed about the protection of their personal data and verbal consent was obtained in consensus with the Spanish Legislation [40]. The six-member panel (four females and two males) underwent three training sessions of two hours each before the evaluation [41]. The trained panelists were presented with three reformulated samples along with the control and the intensity of the sensory parameters was recorded. The sensory evaluation was conducted in infrared-light, temperature and humidity-controlled booths [42]. An unbalanced design was used to determine the allocation of samples randomly and in a random order to each panelist. The intensity of sensory attributes such as general odor, color and odor pertaining to meat or plant of the HPP patties (raw) were noted. Additionally, the intensity of firmness, juiciness, fatness, friability and aftertaste of the HPP + SVCOOK patties was evaluated (Table S2). Data were quantified by measuring the distance in a 15-cm unstructured line scale. Results were analyzed based on the methodology used by Gómez et al. [11] with slight modifications.

### 2.11. Data Analysis and Modeling

Descriptive statistics for the physicochemical parameters of the HPP treated and HPP + SVCOOK samples were calculated. Data analysis and modeling was conducted using Minitab software (Minitab^®^ version 19.2020.1, Minitab LLC, State College, PA, USA).

A mixed-effect model was used to study the effect of different formulations on the physicochemical and sensory properties. Replication was augmented as a random effect in studying the physical parameters. A multiple comparison test was conducted using post hoc Tukey analysis at a 95% confidence interval (*p* < 0.05). The mixed effect model was used in the reformulated patties to identify the significant effect of the fixed terms (protein matrix and fat formulation) and their interaction with the physical and sensory parameters.

A Principal Component Analysis (PCA) was performed on the physicochemical parameters of the samples (meat-based, hybrid and plant-based patties). Two principal components were extracted to retain the total variance of physicochemical parameters and the factor scores for samples were obtained by the regression method. The sample scores were plotted together to explore relationships. The PCA was carried out using the SPSS Statistics software version 27.0 (IBM Corp., Armonk, NY, USA).

## 3. Results and Discussions

### 3.1. Characterization of the Emulsions

The apparent viscosity of the emulsions was recorded as 3.44 and 3.61 Pa·s for the soya and hydro-gelled emulsions, respectively. The *hy* emulsion comprised of a single color component (100%), whereas the *so* emulsion comprised 5% of a second color component. The color values and the pH of the emulsions are presented in Table S1.

### 3.2. Proximate Analysis of Samples

The proximate analysis and pairwise grouping of the samples are summarized in Table 1. It was noted that the meat patties (M) formulated with pork backfat (*ba*) had almost 22 g/100 g fat content after HPP and HPP + SVCOOK treatments, whereas the fat replaced formulations had an average fat content of 8.4 g/100 g. The patty samples had a protein content varying from 20–17 g/100 g post treatments. The patties prepared with the *so* emulsion had higher protein content due to the addition of SPI in the emulsion. Due to the lower fat content in the samples, the fat replaced patties can be labeled as “reduced fat content,” according to The Council of the European Union [43]. Similar reduced fat content was reported in previous fat replacement studies because the emulsions have lesser fat content compared to animal fats generally used as the reference [17,44,45]. Fat replacement led to an increase in moisture and ash contents due to the higher percentage of water present in the emulsions used [46].

### 3.3. Physical Parameters of HPP Treated Patties

#### 3.3.1. Weight Loss and pH

The pH of the HPP treated samples varied significantly (*p* < 0.05) based on the protein matrix used. Maximum values were obtained in the P samples (6.20–6.21) followed by H (6.01–6.02) and M (5.67–5.76) samples, as Table 1 shows. The protein matrix, the emulsion used and their interactions were found to have a significant effect (*p* < 0.05) on the pH of the samples. Results with no effect of the emulsion on the pH of the reformulated patties were previously reported [46,47]. Contrastingly, significantly lower pH was seen in beef patties prepared with cocoa bean shell flour and walnut oil-based emulsion [44].

A significant difference was noted among the weight loss of M-*ba* and M-*hy* patties. It stands out that the M patties formulated with the hydro-gelled emulsion (M-*hy*) had the maximum weight loss (2.98 g/100 g), followed by the other patties prepared with the *hy* emulsion, H-*hy* (2.08 g/100 g) and P-*hy* (2.0 g/100 g). The patties manufactured with *so* emulsion had lower weight losses (0.90–1.96 g/100 g), with H patties exhibiting the lowest values. The protein matrix and the emulsion used imparted significant effects (*p* < 0.05) on the weight loss of the patties, although no significant effect of the interaction terms was found.

#### 3.3.2. Instrumental Color

The luminosity (L*) of the HPP treated M and H protein matrix had similar values (47.60–53.32) to the M-*ba* (51.78) samples. Nevertheless, in the P samples, a significant difference (*p* < 0.05), as well as the highest values (61.92–63.77), were observed in this coordinate (L*). Similarly, there were no statistically significant differences (*p* > 0.05) between the redness (a*) of M and P samples (9.10–10.84) compared to the reference (M-*ba*; 8.83). Contrastingly, a significant difference in the a* (*p* < 0.05) of P samples was noted and it also had the highest values (13.37–13.97). A reduction in the a* value has been previously reported in high pressure treated meat samples due to the oxidation of the ferrous myoglobin to ferric myoglobin [30,48]. The M protein matrix with both emulsions (12.26–14.71) had similarity with M-*ba* (13.66) in yellowness (b*), whereas the H and P samples (19.97–27.83) were significantly different (*p* < 0.05). These results are shown in Table 2.

Color values mainly depended on the protein matrix (*p* < 0.05) rather than the fat formulation used. There was no significant interaction (*p* > 0.05) between the fat formulation used and the protein matrix in the fat replaced patties. Barros et al. [49] reported similar findings with no significant difference in the fat replaced beef patties (algal and wheat germ oil emulsions) because of the fat used. Until 60%, replacement of pork backfat with hydro-gelled emulsion (chia and linseed oil) also reported no significant difference in the color of raw beef patties (*Rectus femoris*). Contrary to our findings, significant differences in the L* and the b* values of raw pork patties formulated with hydrogels of walnut and pistachio oil compared to pork backfat were reported by Foggiaro et al. [47]. Higher L*, a* and b* values were noted in bologna-type sausage prepared with gelled emulsion due to the varying oil globule size in animal and emulsion leading to different reflectance properties [32]. Botella-Martinez et al. [44] meanwhile noticed similar findings. The color values of beef patties prepared with tiger nut oil emulsion also presented increased color values [50]. The varying results might be attributed to the composition of the emulsion used and its interaction with the protein matrix [44,50].

#### 3.3.3. Instrumental Texture

The hardness of the HPP subjected M-*so* (7.06 N) and P-*hy* (8.27 N) was not statistically different from the M-*ba* samples (8.15). The P-*so* patties obtained the highest hardness (11.00 N) and H-*hy* the lowest (4.46 N). A significant effect (*p* < 0.05) of the protein matrix and emulsion used was noted in the hardness of the fat replaced patties. No significant difference (*p* > 0.05) was observed between the springiness of M-*ba* (0.52) and neither hybrid (H) or plant (P) protein matrix with *so* emulsion (0.46 and 0.53, respectively). As for the cohesiveness, M-*so* (0.41) and P-*so* (0.34) resembled reference (M-*ba*; 0.39) values. The cohesiveness of all the reformulated M and H patties was similar irrespective of the emulsions used, with the highest (0.41–0.64) and lowest (0.31–0.33) values, respectively. All the factors and their interactions were significant while comparing the cohesiveness of the fat replaced samples. H-*so* (1.03) and P-*so* (1.96) had similar chewiness to M-*ba* (1.61 N). Moreover, no significant effect (*p* > 0.05) of the emulsions used on chewiness was noted, but simultaneous significant interaction (*p* < 0.05) between the protein matrix and emulsions used were seen. These results are collected in Table 3.

Varying results were previously reported with both no effect and significant effect of fat replacement in cooked reformulated meat products which was mainly attributed to the properties of the ingredients used in the preparation [11,12,23,46,47]. Partial replacement of native fat by konjac gel in sausages was found to reduce the hardness and simultaneously increase cohesiveness. There was no significant effect on either springiness or chewiness. The reduction in hardness of the sausages was the inference of the higher moisture–protein ratio in the product [16]. Conversely, the formation of harder structures with the reduction in fat was reported by other researchers [20].

#### 3.3.4. Principal Component Analysis

**Figure 2:**
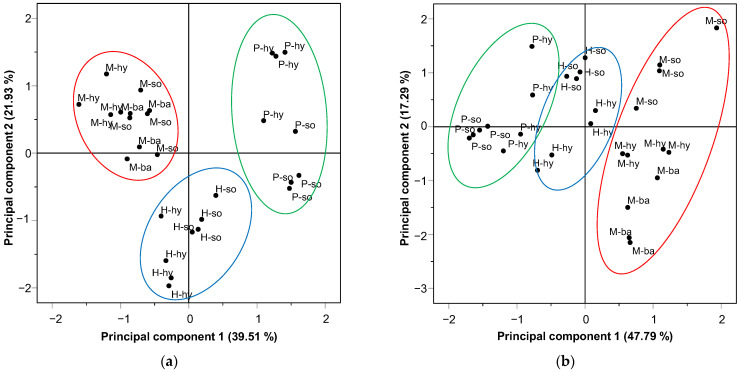
Plotting principal component for samples treated by HPP (**a**) or HPP + SVCOOK (**b**) according to protein matrix (M = meat-based patty; H = hybrid patty; P = plant-based patty) and fat replacement (*ba*: backfat; *hy*: hydrocolloid emulsion; *so*: soya emulsion).

The initial information obtained has been reduced to two principal components, explaining 64.44% of the total variance (Figure 2a). All physicochemical parameters and hardness, L* and b*, were associated with the first principal component (PC1). The remaining textural parameters and a* were associated with the second principal component (PC2). The PC1 factor (39.51% variance) makes a clear difference between M patties from P patties, having the opposite behavior. The H patties, as expected, resemble both protein bases. Nevertheless, the PC2 (21.93% variance) helped to differentiate the hybrid patties from the others.

Finally, the different lipids in the M patties reflect a more homogeneous behavior (in both principal components). The opposite happens in H and P patties. The PC2 factor (Figure 2a) shows a clear difference between the replaced fats (*hy* and *so* emulsions).

### 3.4. Physical Parameters HPP + SVCOOK Treated Patties

#### 3.4.1. Weight Loss and pH

It was noted that the HPP + SVCOOK treated samples had a significantly different (*p* < 0.05) pH, as Table 1 shows. Similar values were observed only for reference (5.68) and M-*hy* (5.71). The plant-based samples had the highest values (6.14–6.19); as expected, the H samples had intermediate values (5.80–5.96).

Regarding weight loss, there were significant differences (*p* < 0.05) between M-*ba* (8.25 g/100 g) and the new formulations. The M patties had the highest (13.84–16.34 g/100 g) weight loss, followed by the M-*ba* patties. The minimum value for cooking loss was observed in the P-*so* patties (2.00 g/100 g).

A significant effect (*p* < 0.05) of the protein matrix and the emulsion on the pH and cooking loss was noted. A similar effect of fat replacement (hydro-gelled emulsion–chia and linseed oils) on pH was reported by other researchers [12]; conversely, no effect of cooking loss was noticed. A relevant increase in cooking loss was reported when fat replacement (linseed oil–olive oil emulsion) was conducted by Gómez et al. [11]. However, the cooking loss was lower in beef burger patties prepared with a cocoa bean shell and walnut oil-based emulsion, which might be due to hydrogen bonds between meat compounds and water leading to higher water retention [44]. Simultaneously a low pH was also noted in the samples. Barros et al. [50] reported similar cooking loss results in beef burgers manufactured with tiger nut oil emulsion. Instead, no significant effect of alginate-based hydrogels fat replacer was noticed in foal burgers [46].

#### 3.4.2. Instrumental Color

Color value trends in HPP + SVCOOK samples were found to be similar to the trends observed in the HPP treated samples, specifically in L* and b*. M and H patties (48.81–55.90) had similar behavior to M-*ba* (50.53) in L* coordinate. A significant difference (*p* < 0.05) in the L* of P samples, as well as the highest values (60.80–62.50), was detected. In the b* coordinate, there were no significant differences (*p* > 0.05) between M (12.60–13.97) and M-*ba* (12.55). However, a significant difference (*p* < 0.05) was shown in H and P samples (19.82–29.17) compared to the reference, with P samples obtaining the highest values. The a* coordinate of M-*ba* (8.73), M (8.81–9.68) and P-*so* (9.67) exhibited identical values. A significant difference (*p* < 0.05) was noted with the rest of the samples (10.27–11.75), focusing on H samples with the highest values. The results can be observed in Table 2.

The protein matrix alone had a significant effect on the L*, a* and b* values of the patties. Conversely, reformulated cooked (electric grill–core temperature of 72 °C) beef patties (*Rectus femoris*), 40–100% pork backfat substituted (chia–linseed oil hydro-gelled emulsion) presented significant differences in color values compared to the control samples with 100% pork backfat according to Heck et al. [12]. Significantly lower color values were detailed in beef patties elaborated with fat replacers (cocoa shell flour and walnut oil-based emulsion), which might be due to the color and composition of oil and emulsifying agents used and their interaction with other components [44]. Similarly, Cittadini et al. [46] noted a significant difference in fat replaced foal meat patties.

#### 3.4.3. Instrumental Texture

The hardness of the HPP + SVCOOK treated H-*so* had similar values to the M-*ba* samples. The highest hardness was recorded in the M-*so* patties (34.29 N), whereas the P-*so* patties had the lowest values (13.11 N). Contrary to our finding, reformulated beef patties had lower hardness than the control when they were cooked on a hot plate griddle [11]. The H patties prepared with both the emulsions and the P-*hy* patties had similar springiness as seen in the M-*ba* and M-*so* samples (0.71–0.78).

The chewiness of the H-*so* had similar values as the M-*ba* and M-*hy* patties (8.95–11.05 N). The maximum chewiness values were observed in the M-*so* samples (16.54 N). It was noted that all the texture parameters of the reformulated patties were significantly affected (*p* < 0.05) by the protein matrix, emulsions used and their interaction (Table 3).

Other researchers have noted an increase in all the texture parameters of cooked pork patties except for springiness when pork backfat is replaced with hydrogels of walnut and pistachio oil [47]. Similar results were reported by Barros et al. [49] in cooked beef patties formulated with algal and wheat germ oil emulsions. Some studies have found no significant difference in the hardness, chewiness and cohesiveness of control and fat replaced patties [50,51]. Cittadini et al. [46] reported no effect on texture parameters except on chewiness when alginate-based hydrogels were used as fat replacers in foal burgers. The results obtained were attributed to the interaction between the oils and fats with meat and their characteristic physicochemical behaviors.

#### 3.4.4. Principal Component Analysis

Analogous to the samples treated by HPP, it has been reduced to two main components. In this case, the explained information reaches 65.08% of the total variance (Figure 2b). All textural parameters, as well as L*, b*, pH, weight loss, ash and moisture contents, were associated with the first principal component (PC1). Protein, fat contents, as well as a* values, were associated with the second principal component (PC2). The PC1 (47.79% variance) reflects, as in the samples treated by HPP, an opposite behavior when comparing the samples with meat-based protein (M) and plant-based protein (P). Instead, the samples with hybrid protein (H) resemble both. It should be noted that the PC2 factor (17.29% variance) helped to differentiate the fats used (*ba*, *hy* and *so* formulations) (Figure 2b).

Finally, it should be noted that the samples treated by HPP + SVCOOK presented a greater homogeneity with respect to the samples treated only with HPP.

### 3.5. Sensory Analysis

#### 3.5.1. HPP Patties

**Figure 3:**
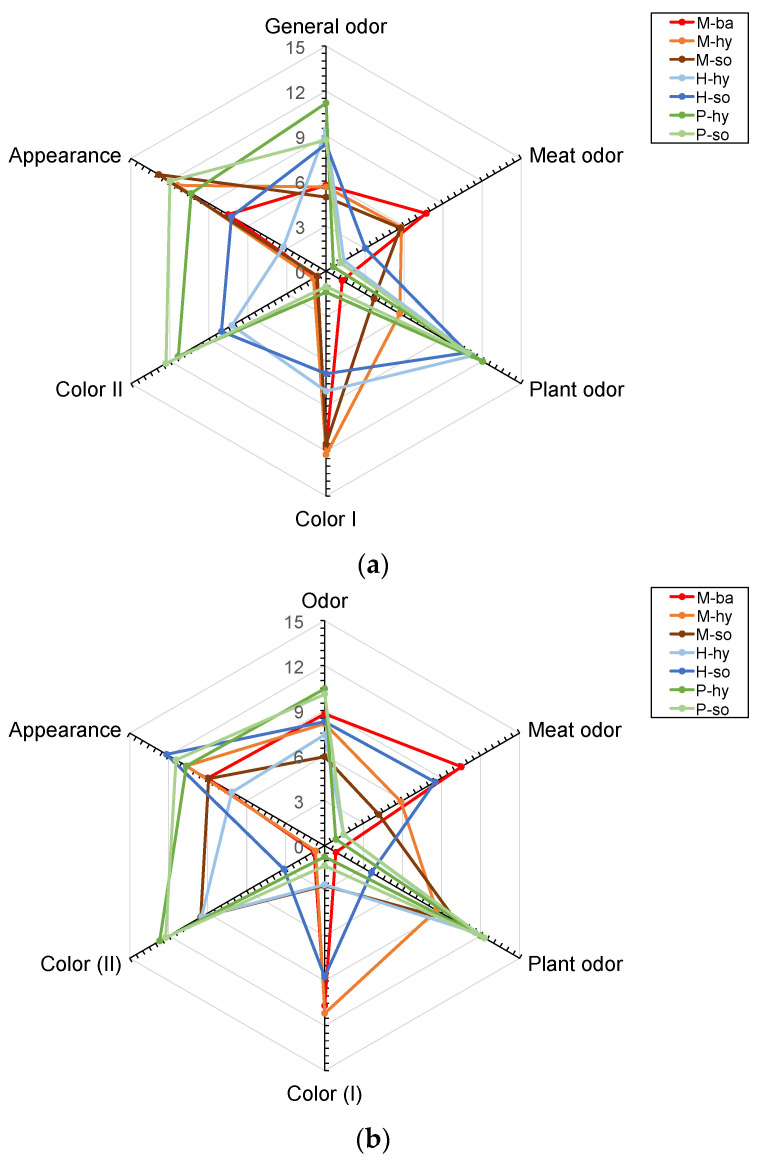

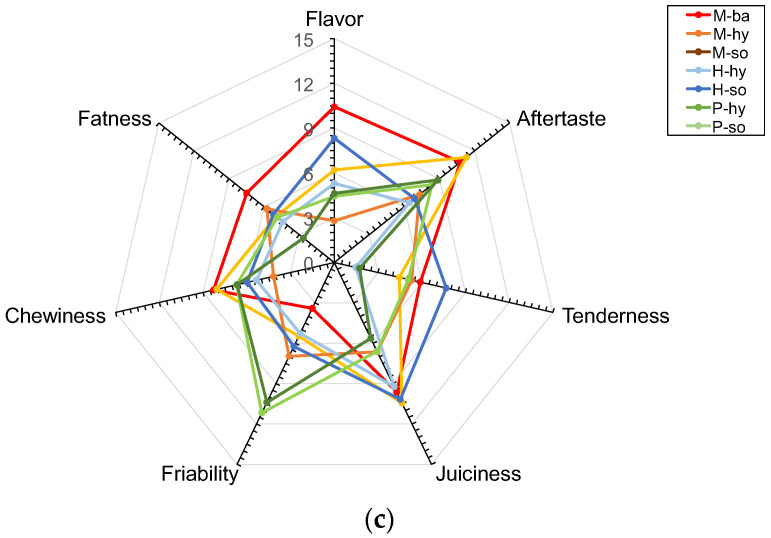
Sensory profile of samples treated by HPP (**a**) or HPP + SVCOOK (**b**,**c**), according to protein matrix (M = meat-based patty; H = hybrid patty; P = plant-based patty) and fat replacement (*ba*: backfat; *hy*: hydrocolloid emulsion; *so*: soya emulsion.

In the uncooked samples, no significant effects (*p* > 0.05) were detected in general odor, meat odor and appearance, as seen in Figure 3a. As for the general odor, M patties had the lowest values and H and P had the highest values. Likewise, the patties with *hy* emulsion had higher values compared to *so* emulsion in the same protein matrix.

Regarding the meat odor, as expected, M samples had the highest values and P samples had the lowest. In addition, H patties obtained intermediate values. On the other hand, significant effects (*p* < 0.05) were detected in the rest of the attributes, as shown the Figure 3a. In plant odor, P and H patties had the highest values and M samples the lowest, as expected. Finally, in color I (color intensity referred to meat) and II (color intensity referred to the plant-based), a similar trend as observed in odor was seen, but in both cases, with significant effects. As expected, H had an intermediate behavior. It can be seen that the samples with meat protein presented a greater intensity in color I and less in color II.

It was noticed that there was no evident difference in the general odor. In contrast, for the intensity of meat odor, a significant difference was observed when computing the mixed effect of the factors, but with the pairwise analysis no significant difference between each formulation could be seen. A significant effect of the use of the emulsion was noted in the assessment of color corresponding to meat. A higher hedonic score and sensory acceptance were previously reported by Poyato et al. [23] in raw fat replaced pork-beef patties (carrageenan based gelled emulsion). Foal meat burgers prepared with partial fat replacement (algal oil-based hydrogels) exhibited no significant difference compared to control burgers [46]. The use of carrageenan-based emulsion in sausages did not affect the sensory characteristics compared to traditional emulsion sausages [32]. Similarly, konjac-based fat replacers did not induce any change in the appearance of dry fermented sausages [16].

#### 3.5.2. HPP + SVCOOK Patties

In the HPP + SVCOOK samples, no significant effects (*p* > 0.05) were detected in general odor, appearance, flavor, firmness, juiciness, chewiness and fatness, as seen in Figure 3b,c. The general odor of the P protein matrix had the highest intensity, followed by M and H samples, respectively. The appearance and firmness showed similar trends. In both cases, M and P samples had similar, higher values than H. M patties obtained the highest values for juiciness and chewiness, followed by H and P patties, respectively. In fatness, the reference and reformulation samples exhibited similar behavior. The M-*ba* patties had the highest flavor, preceded by M, P and H samples, respectively.

On the other hand, significant differences (*p* < 0.05) were detected in the other attributes, as shown in Figure 3b,c. The color attributes (color I and color II) showed a similar trend as observed in the HPP samples. Considering the two odor attributes, only M-*so* had similar values as the reference (M-*ba*). M-*ba* and M-*so* had the highest intensity in meat odor and lowest in plant odor. Regarding the aftertaste, there was a significant difference (*p* < 0.05) between H-*hy* and the other samples. M and H patties had similar friability as the reference (M-*ba*). In contrast, the P samples had the lowest, which easily crumbled under force.

Color responses corresponding to meat color exhibited a significant difference based on the protein matrix; the mix proved to be more like the meat samples when prepared with *so* emulsion and the plant-based samples. The intensity of odor was as expected based on the protein matrix in the formulation adhering to either meat or plant-based. In these variables, M patties obtained a higher valuation in the attributes linked to meat (intensity meat odor and color I) and lower in the attributes related to plant characteristics (intensity plant odor and color II). Likewise, plant protein patties have a completely opposite behavior. In general, it has been seen that samples with hybrid protein are more similar (a greater homogeneity) to the plant-based patties. A significant effect due to the use of different emulsions was only visible in assessing the intensity of meat color. Moreover, it was noted that the interaction between protein and emulsion was significant in the firmness of the HPP + SVCOOK sensory profile. Hanula et al. [52] mentioned significant differences in the trained panel sensory analysis of reformulated beef patties (hydrogel enriched with acai oil). At the same time, Poyato et al. [23] reported no significant difference in the sensory profile of reformulated pork-beef patties with carrageenan based gelled emulsion. In similar studies with partially fat replaced Bologna-type sausages, the panelists could not distinguish between the control and the reformulated samples [32]. Cittadini et al. [46] have reported a significant difference in flavor alone of cooked foal burgers prepared with algal oil-based hydrogel as a partial fat replacer. Beef burgers elaborated with cocoa bean shell flour and walnut oil-based emulsion (50% and 100% replacement) showed characteristically higher color and hardness scores. At the same time, lower acceptability scores were reported for the 100% fat replaced samples [44].

## 4. Conclusions

The difference in physicochemical and sensory parameters of HPP or HPP + SVCOOK treated patties based on the fat used was the least. The texture parameters of the HPP + SVCOOK hybrid patties, irrespective of the emulsion used, behaved similarly to the meat patties prepared with pork backfat. Prominent differences in the sensory profile of the HPP treated formulations were evident but, interestingly, minimal differences between HPP + SVCOOK formulations and reference were noted (except for color and odor pertaining to meat or plant-based, aftertaste and friability). No characteristic differences between the reformulated HPP + SVCOOK patties were observed except in color pertaining to meat color. It could be concluded that a successful fat replacement using either one of the soya or hydro-gelled emulsion could be conducted in HPP + SVCOOK patties. Future research on the fatty acid profiling, lipid oxidation, market research and nutritional advantages of the sustainable, innovative and fat replaced patties HPP + SVCOOK patties needs to be performed.

## Figures and Tables

**Table 1:** Physicochemical characteristics of samples treated by HPP or HPP + SVCOOK according to protein matrix (M: meat-based patty; H: hybrid patty; P: plant-based patty) and fat replacement (*ba*: backfat; *hy*: hydro-gelled emulsion; *so*: soya emulsion). Values are expressed as mean (standard deviation).

Treatment	Sample	Weight Loss (g/100 g)	pH	Moisture (g/100 g)	Fat (g/100 g)	Protein (g/100 g)	Ash (g/100 g)
HPP	M-*ba*	1.07 (0.08) ^b^	5.70 (0.06) ^cd^	58.4 (0.3) ^c^	22.2 (0.7) ^a^	17.3 (0.2) ^c^	2.3 (0.2) ^a^
	M-*hy*	2.98 (0.59) ^a^	5.67 (0.02) ^d^	69.0 (1.1) ^a^	10.6 (0.4) ^b^	17.0 (0.4) ^c^	3.3 (1.0) ^ab^
	M-*so*	1.96 (1.04) ^ab^	5.76 (0.04) ^c^	69.1 (0.1) ^a^	9.7 (0.4) ^c^	19.6 (1.1) ^ab^	2.5 (0.1) ^bc^
	H-*hy*	2.08 (0.50) ^ab^	6.02 (0.02) ^b^	62.2 (0.6) ^b^	10.3 (0.2) ^bc^	17.6 (0.1) ^c^	3.0 (0.1) ^abc^
	H-*so*	0.90 (0.45) ^b^	6.01 (0.04) ^b^	62.6 (0.4) ^b^	7.9 (0.4) ^d^	19.6 (0.2) ^ab^	3.0 (0.1) ^abc^
	P-*hy*	2.09 (0.47) ^ab^	6.20 (0.02) ^a^	55.7 (0.2) ^d^	8.3 (0.4) ^d^	18.8 (0.6) ^d^	3.7 (0.1) ^a^
	P-*so*	1.23 (0.31) ^b^	6.21 (0.06) ^a^	56.6 (0.1) ^d^	6.0 (0.2) ^e^	20.1 (0.2) ^a^	3.6 (0.1) ^a^
HPP + SVCOOK	M-*ba*	8.25 (1.33) ^b^	5.68 (0.03) ^e^	57.6 (0.9) ^cd^	22.8 (2.1) ^a^	17.6 (0.8) ^c^	2.3 (0.1) ^ab^
	M-*hy*	16.34 (3.20) ^a^	5.71 (0.01) ^e^	68.1 (1.6) ^a^	9.6 (1.2) ^b^	18.4 (0.2) ^bc^	3.3 (0.7) ^a^
	M-*so*	13.84 (2.11) ^a^	5.80 (0.04) ^d^	67.8 (0.7) ^a^	9.1 (0.6) ^b^	20.3 (0.4) ^a^	1.6 (1.5) ^b^
	H-*hy*	3.71 (0.72) ^c^	5.96 (0.05) ^c^	61.9 (0.8) ^b^	10.0 (0.4) ^b^	17.6 (0.4) ^c^	3.1 (0.0) ^ab^
	H-*so*	2.00 (0.50) ^c^	6.02 (0.01) ^b^	63.0 (0.1) ^b^	7.8 (0.2) ^bc^	19.7 (0.3) ^ab^	3.1 (0.0) ^ab^
	P-*hy*	2.56 (1.89) ^c^	6.14 (0.03) ^a^	55.6 (1.2) ^d^	8.5 (1.3) ^b^	18.7 (1.5) ^abc^	3.7 (0.0) ^a^
	P-*so*	0.86 (0.05) ^c^	6.19 (0.02) ^a^	58.0 (1.4) ^c^	5.3 (1.0) ^c^	19.5 (0.4) ^ab^	3.6 (0.2) ^a^

Different superscripts in the same column indicate significant differences (*p* < 0.05) by the Tukey test. Comparisons were within the same treatment.

**Table 2:** Color parameters of samples treated by HPP or HPP + SVCOOK according to protein matrix (M: meat-based patty; H: hybrid patty; P: plant-based patty) and fat replacement (*ba*: backfat; *hy*: hydro-gelled emulsion; *so*: soya emulsion). Values are expressed as mean (standard deviation).

Treatment	Sample	L*	a*	b*
HPP	M-*ba*	51.78 (4.18) ^bc^	8.83 (1.59) ^b^	13.66 (4.38) ^d^
	M-*hy*	47.60 (3.12) ^c^	9.10 (2.51) ^b^	12.26 (4.78) ^d^
	M-*so*	50.07 (5.41) ^bc^	10.84 (1.91) ^b^	14.71 (5.45) ^cd^
	H-*hy*	52.88 (4.64) ^bc^	13.37 (2.39) ^a^	19.97 (5.49) ^bc^
	H-*so*	53.32 (6.54) ^b^	13.97 (2.59) ^a^	20.88 (5.66) ^b^
	P-*hy*	61.92 (4.31) ^a^	10.38 (0.93) ^b^	27.83 (2.89) ^a^
	P-*so*	63.77 (3.62) ^a^	9.90 (0.73) ^b^	26.97 (3.79) ^a^
HPP + SVCOOK	M-*ba*	50.53 (4.50) ^cd^	8.73 (0.64) ^d^	12.55 (5.08) ^c^
	M-*hy*	48.81 (4.89) ^d^	8.81 (1.11) ^d^	12.60 (5.28) ^c^
	M-*so*	50.71 (4.46) ^cd^	9.68 (0.82) ^cd^	13.97 (5.49) ^bc^
	H-*hy*	53.94 (4.87) ^cd^	11.75 (1.71) ^a^	19.82 (7.09) ^b^
	H-*so*	55.90 (4.25) ^bc^	11.36 (1.06) ^ab^	19.82 (5.77) ^b^
	P-*hy*	60.80 (5.81) ^ab^	10.27 (0.49) ^bc^	29.17 (4.01) ^a^
	P-*so*	62.50 (4.79) ^a^	9.67 (0.59) ^cd^	26.78 (3.34) ^a^

Different superscripts in the same column indicate significant differences (*p* < 0.05) by the Tukey test. Comparisons were within the same treatment.

**Table 3:** Textural parameters of samples treated by HPP or HPP + SVCOOK according to protein matrix (M: meat-based patty; H: hybrid patty; P: plant-based patty) and fat replacement (*ba*: backfat; *hy*: hydro-gelled emulsion; *so*: soya emulsion). Values are expressed as mean (standard deviation).

Treatment	Sample	Hardness (N)	Springiness	Cohesiveness	Chewiness (N)
HPP	M-*ba*	8.15 (1.79) ^b^	0.52 (0.06) ^bc^	0.39 (0.06) ^bc^	1.61 (0.28) ^bc^
	M-*hy*	5.95 (2.26) ^bc^	0.67 (0.05) ^a^	0.46 (0.02) ^a^	1.84 (0.70) ^ab^
	M-*so*	7.06 (1.02) ^b^	0.66 (0.05) ^a^	0.41 (0.03) ^ab^	1.92 (0.31) ^ab^
	H-*hy*	4.46 (0.94) ^c^	0.45 (0.06) ^d^	0.31 (0.03) ^d^	0.60 (0.14) ^d^
	H-*so*	6.74 (1.27) ^bc^	0.46 (0.05) ^cd^	0.33 (0.03) ^d^	1.03 (0.26) ^cd^
	P-*hy*	8.27 (2.28) ^b^	0.67 (0.04) ^a^	0.43 (0.05) ^ab^	2.41 (0.82) ^a^
	P-*so*	11.00 (3.18) ^a^	0.53 (0.06) ^b^	0.34 (0.03) ^cd^	1.96 (0.59) ^ab^
HPP + SVCOOK	M-*ba*	29.90 (7.00) ^ab^	0.71 (0.06) ^b^	0.51 (0.05) ^bc^	11.05 (3.37) ^bc^
	M-*hy*	18.81 (4.44) ^cd^	0.81 (0.06) ^a^	0.58 (0.09) ^ab^	8.95 (2.97) ^bc^
	M-*so*	34.29 (9.84) ^a^	0.78 (0.02) ^ab^	0.61 (0.03) ^a^	16.54 (5.18) ^a^
	H-*hy*	13.35 (6.38) ^d^	0.72 (0.06) ^b^	0.48 (0.07) ^c^	5.01 (3.30) ^cd^
	H-*so*	23.38 (4.61) ^bc^	0.76 (0.02) ^ab^	0.50 (0.04) ^bc^	9.05 (2.25) ^bc^
	P-*hy*	16.57 (8.10) ^cd^	0.73 (0.09) ^b^	0.50 (0.10) ^bc^	6.85 (4.67) ^cd^
	P-*so*	13.11 (3.69) ^d^	0.57 (0.08) ^c^	0.33 (0.02) ^d^	2.38 (0.55) ^d^

Different superscripts in the same column indicate significant differences (*p* < 0.05) by the Tukey test. Comparisons were within the same treatment.

## Data Availability

Data is contained within the article and Supplementary Material.

## Supplementary Materials

The following supporting information can be downloaded at: https://www.mdpi.com/article/10.3390/foods11223678/s1, Table S1: Physical parameters of emulsions used in the manufacture of patties. Values are expressed as mean (standard deviation); Table S2: Description of attributes used in the sensory evaluation of samples treated by HPP or HPP + SVCOOK (continuous scale with anchor words; from 0 “very low intensity” to 15 “very high intensity”).
