# Supplementary material for: Comparison of High Hydrostatic Pressure Processed Plus Sous-Vide Cooked Meat-Based, Plant-Based and Hybrid Patties According to Fat Replacement
Source: Foods. 2022 Nov 17;11(22):3678. doi: 10.3390/foods11223678 (PMC9689922; doi:10.3390/foods11223678)
Supplement: Supplementary file 1 [file foods-11-03678-s001.zip › foods-2018915-SupplementaryMaterials.pdf]

Supplementary Materials

Table S1. Physical parameters of emulsions used in the manufacture of patties. Values are expressed as mean (standard deviation).

Parameter	Hydrogelled emulsion	Soya emulsion
pH	6.61 (0.08)	7.11 (0.05)
*L**	73.34 (2.95)	83.56 (0.09)
*a**	11.94 (3.26)	1.13 (0.44)
*b**	79.10 (3.26)	25.93 (0.07)

Table S2. Description of attributes used in the sensory evaluation of samples treated by HPP or HPP+SVCOOK (continuous scale with anchor words; from 0 “very low intensity” to 15 “very high intensity”).

Attribute	Description
**General odor**	Olfactory sensation perceived through the olfactory organ (external via) when smelling the patty
**Meat odor**	Olfactory sensation perceived using a raw meat patty as a reference
**Plant odor**	Olfactory sensation perceived taking as reference a patty manufactured with cereals and legumes
**Color I**	Visual sensation of hue, saturation and clarity induced by stimulation of the retina by light waves of various wavelengths (the reference being the brown color of cooked meat patty)
**Color II**	Visual sensation of hue, saturation and clarity induced by stimulation of the retina by light waves of various wavelengths (the reference being the yellow color of soft nougat)
**Appearance**	Visual property of patty surface considering the ratio of meat/fat
**Flavor**	The complex combination of olfactory, gustatory and trigeminal sensations perceived during the tasting (the reference being the conventional meat patty)
**Tenderness**	Force needed to compress a cooked meat patty between the molars
**Juiciness**	The sensation of moisture in the mouth when biting the cooked patty 5 times
**Friability**	The ease with which the cooked patty breaks into small fragments after chewing
**Chewiness**	Time required (in seconds) to grind a cooked patty in the mouth at a constant speed so that it reaches a suitable consistency for swallowing
**Fatness**	The fat sensation that occurs in the oral cavity after the removal of the cooked patty
**Aftertaste**	Olfactory and/or gustatory sensations that occur after removal of the cooked patty and differ from the sensation perceived while the product was in the mouth
